# Supplementary material for: Serum Metabolites Associated with Muscle Hypertrophy after 8 Weeks of High- and Low-Load Resistance Training
Source: Metabolites. 2023 Feb 24;13(3):335. doi: 10.3390/metabo13030335 (PMC10058868; doi:10.3390/metabo13030335)
Supplement: Supplementary file 1 [file metabolites-13-00335-s001.zip › metabolites-2132909-supplementary.pdf]

Table 1. Dietary intake

Protocol	Kcal		CHO (g)		PRO (g)		LIP (g)	
	Pre	Pos	Pre	Pos	Pre	Pos	Pre	Pos
HL-RT	1952 ± 548	2098 ± 807	246 ± 96	261 ± 111	88 ± 32	92 ± 56	64 ± 33	74 ± 52
LL-RT	1836 ± 586	1785 ± 454	225 ± 97	210 ± 71	90 ± 36	101 ± 36	61 ± 24	55 ± 23

Kcal = total caloric consumption, CHO = carbohydrate consumption, PRO = protein consumption, LIP = lipid consumption, HL-RE = high-load resistance training, LL-RT = low-load resistance training, g = grams, Pre = day before the first training session, Pos = day before the last training session.

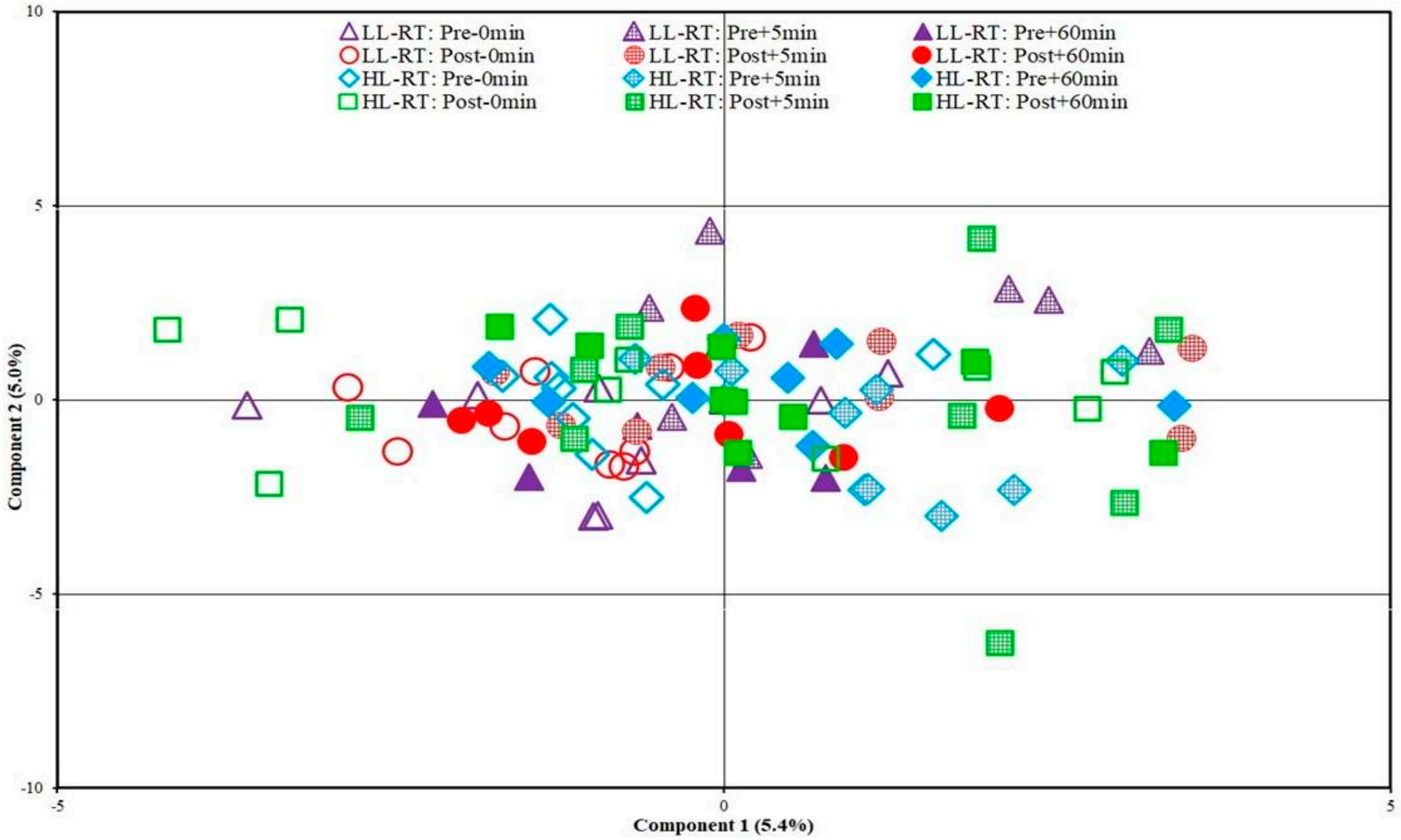

Figure1. Principal Component Analysis (PCA).
